# Burden of chronic obstructive pulmonary disease in China from 1990 to 2021: a population-based study

**DOI:** 10.3389/fmed.2025.1674952

**Published:** 2025-10-15

**Authors:** Xuefang Wang, Jing Liu, Juan Li, Ling Zhang, Xiuqin Zeng, Ruijun Cai

**Affiliations:** Department of Pharmacy, Shanghai General Hospital Jiuquan Hospital (The People’s Hospital of Jiuquan), Jiuquan, Gansu, China

**Keywords:** chronic obstructive pulmonary disease, China, incidence, prevalence, mortality, DALYs

## Abstract

**Background:**

Chronic obstructive pulmonary disease (COPD) is associated with high prevalence, disability, and mortality rates. As one of the most populous countries facing a significant burden of COPD, the extent of this burden in China remains inadequately defined. This study utilizes data from the Global Burden of Disease Study 2021 (GBD 2021) to analyze the current status and trends of COPD burden in China, aiming to provide epidemiological data to support prevention, early intervention, and policy formulation related to COPD.

**Methods:**

This study employs the GBD 2021 database and utilizes various statistical methods, including estimated annual percentage change, Joinpoint regression analysis, decomposition analysis, predictive analysis, and risk factor attribution, to conduct a stratified analysis of the burden of COPD in China by sex, age, and time.

**Results:**

Between 1990 and 2021, the age-standardized rate (ASR) of COPD in China showed a significant decline, with projections indicating that this trend will continue. However, the rate of decline has slowed in recent years, and the absolute number of COPD patients continues to rise. In 2021, the age-standardized incidence rate (ASIR) was 215.62 per 100,000, the age-standardized prevalence rate (ASPR) was 2,499.35 per 100,000, the age-standardized mortality rate (ASMR) was 73.23 per 100,000, and the age-standardized disability-adjusted life years (ASDR) was 1,227.66 per 100,000. The decline was more pronounced in females than in males, with females experiencing a lower overall burden. For both sexes, the ASR of COPD increased with age; prior to age 60, rates were similar, but after age 60, males exhibited higher ASMR and ASDR than females. Population aging is identified as the primary driver of the increasing burden of COPD, while epidemiological changes contribute to its reduction. Smoking remains the leading risk factor for mortality, with eight related risk factors identified.

**Conclusion:**

While the ASR of COPD in China has significantly improved, the absolute burden continues to escalate, with notable differences by age and sex. Future efforts should focus on enhancing preventive measures for major risk factors and implementing early screening for high-risk populations to promote early diagnosis and treatment strategies, thereby alleviating the disease burden.

## 1 Background

Chronic obstructive pulmonary disease (COPD) is a common chronic respiratory condition characterized by persistent airflow limitation and chronic airway inflammation. Due to its high prevalence, significant disability burden, elevated mortality rates, and prolonged disease course, COPD has become one of the most pressing public health issues globally (1–5). The insidious onset and atypical early clinical manifestations of COPD contribute to a low diagnostic rate. It is estimated that approximately 70% of COPD patients worldwide remain undiagnosed, leading to delays in seeking medical attention and disease progression (6). Research indicates that undiagnosed COPD patients face a mortality risk approximately 23% higher than those without airflow limitation (7). Insufficient diagnosis hinders timely intervention, making patients more susceptible to acute exacerbations. About 46% of COPD patients experience at least one acute exacerbation annually, with 19% requiring hospitalization (8). Acute exacerbations not only accelerate the decline in lung function but also severely impair quality of life, significantly increasing the risks of hospitalization, disability, and mortality, thereby exacerbating the burden on both families and healthcare systems.

In the context of a growing global burden, the latest data from the Global Burden of Disease Study 2021 (GBD 2021) reveals that China ranks first in the world for the incidence, prevalence, and mortality of COPD, indicating an extremely heavy disease burden (9). This burden is further exacerbated by a lack of awareness and management deficiencies. Research shows that approximately two-thirds of patients with moderate to severe COPD in China exhibit no obvious clinical symptoms, with awareness rates below 1%. Only 5.9% of patients have undergone pulmonary function testing (such as spirometry), and merely 11.7% have received any form of treatment (10). Concurrently, demographic aging, ongoing population growth, and environmental pollution are expected to further drive the rising trend of COPD burden in China over the coming decades (11, 12).

To optimize the prevention and control strategies for COPD in China, it is essential to gain a deeper understanding of the evolving trends in its disease burden. This study utilizes the GBD 2021 database and employs various statistical methods, including trend analysis, decomposition analysis, predictive modeling, and attribution of risk factors, to systematically assess the changes in the burden of COPD in China from 1990 to 2021 and to forecast its future trajectory. The aim is to provide important evidence-based support for the formulation of precise and sustainable public health interventions.

## 2 Materials and methods

### 2.1 Data sources and disease definition

This study is based on all available data related to COPD in China from the GBD 2021 database, including multi-source data from the China Disease Surveillance System, Cause of Death Registry, national chronic disease epidemiological surveys (such as the China Chronic Disease and Nutrition Surveillance, the China Pulmonary Health Study), and published respiratory disease research. The Global Burden of Disease (GBD) project integrates and estimates these data using a set of standardized modeling methods, including DisMod-MR, spatiotemporal Gaussian process regression, and Bayesian meta-regression. Since the GBD database is an integrated data source, the original literature list is not provided. For a comprehensive methodology and data sources, please refer to the core GBD 2021 paper (13). This study extracted and used all age-specific and sex-specific indicators related to COPD in China from the GBD 2021 database, including incidence, prevalence, mortality, and Disability-Adjusted Life Years (DALYs). These can be found at: https://vizhub.healthdata.org/gbd-results?params=gbd-api-2021-permalink/ec7be55cfb5472a2f931c4f9a18f3e76.

The data selection criteria were as follows: the region was limited to “China,” the disease category was specified as “chronic obstructive pulmonary disease,” risk factors were selected as “most detailed risks,” the gender included “male, female, and overall,” and the time frame was “1990–2021.” To ensure comprehensive age coverage for burden estimation, we selected all standard 5-year age intervals (i.e., <5 years, 5–9 years, …, 95 years and older) from the GBD database for age groups, in order to fully capture the distribution of COPD across the entire lifespan. The diagnosis of COPD was based on the International Classification of Diseases, 10th Revision (ICD-10) codes, including J41, J42, J43, J44, and J47. The extracted indicators included incidence, prevalence, mortality, and disability-adjusted life years (DALYs), with all indicators reported alongside a 95% uncertainty interval (UI). Additionally, all age-standardized rate (ASR) indicators in this study, including Age-Standardized Incidence Rate (ASIR), Age-Standardized Prevalence Rate (ASPR), Age-Standardized Mortality Rate (ASMR), and Age-Standardized DALYs Rate (ASDR), were directly extracted from the final estimates of the GBD 2021 database. In generating these estimates, GBD used its standardized GBD World Standard Population Structure for calculation, aimed at eliminating the influence of regional and temporal population age composition differences, ensuring high comparability of disease burden trends across different countries and globally. China’s population data can be found directly at: https://vizhub.healthdata.org/gbd-results?params=gbd-api-2021-permalink/67a8e77c3f7b6b0e9152f344ef217f96. All data cleaning, statistical analyses, and visualizations were conducted using R software (version 4.4.2), utilizing packages such as dplyr, tidyverse, BAPC, and ggplot2. Statistical significance was set at *P* < 0.05.

### 2.2 Statistical analysis

To assess the temporal trends in the burden of COPD in China from 1990 to 2021, this study employed a combined analysis of the estimated annual percentage change (EAPC) and Joinpoint regression model (14, 15). The formula for calculating EAPC is: EAPC = 100 × [exp(β) − 1], where β represents the linear regression coefficient between the natural logarithm of ASR [ln(ASR)] and year (X): ln(ASR) = α + βX + ε. Joinpoint regression segments the time series by identifying “turning points” in trend changes, calculating the annual percentage change (APC) for each segment and the average annual percent change (AAPC) for the overall trend. EAPC, AAPC, and APC are reported with 95% confidence intervals (CI); if the effect value >0 and the lower limit >0, it indicates an upward trend; conversely, if the effect value <0 and the upper limit <0, it indicates a downward trend.

This study also employed the DasGupta decomposition method to quantify the net changes in the burden of COPD from 1990 to 2021, assessing the independent contributions of population growth, aging, and epidemiological changes to reveal the relative impact of different driving factors on the evolution of COPD burden (16, 17).

Future trend predictions were conducted using the Bayesian Age-Period-Cohort (BAPC) model to evaluate the changes in the burden of COPD in China from 2022 to 2050. The BAPC model utilized a second-order random walk (RW2) prior to smooth the effects of age, period, and birth cohort, combined with nested Laplace approximation to avoid convergence and mixing issues commonly faced in traditional Bayesian analyses using Markov Chain Monte Carlo methods, thereby enhancing predictive accuracy (18).

Additionally, the population attributable fraction (PAF) was calculated to assess the relative contribution of various risk factors to the burden of COPD. PAF represents the proportion of disease burden that could be avoided by completely eliminating a specific exposure, calculated using the formula: PAF = [P × (RR − 1)]/[P × (RR − 1) + 1], where P is the prevalence of the exposure in the population, and RR is the relative risk (or odds ratio, OR) of the exposed group compared to the non-exposed group. P and RR are typically estimated based on population survey data and epidemiological literature.

## 3 Results

### 3.1 Disease burden

**FIGURE 1:**
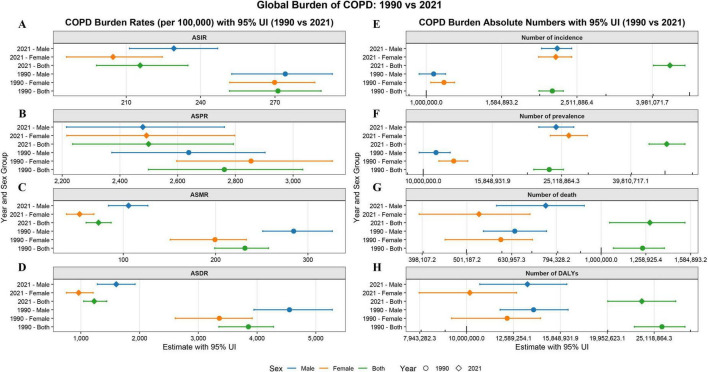
Burden of COPD [**(A)** ASIR; **(B)** ASPR; **(C)** ASMR; **(D)** ASDR; **(E)** Incidence; **(F)** Prevalence; **(G)** Mortality; **(H)** DALYs].

Between 1990 and 2021, the ASR for COPD in China showed significant improvement, although the absolute disease burden continued to rise. The ASIR decreased from 271.22 per 100,000 (95% UI: 251.66–288.62) to 215.62 per 100,000 (95% UI: 198.00–234.90), with an EAPC of −0.84% (95% CI: −0.88%, −0.81%). The decline in women (EAPC: −0.96%, 95% CI: −1.05%, −0.86%) was significantly faster than in men (EAPC: −0.71%, 95% CI: −0.80%, −0.62%). The ASPR decreased from 2761.81 per 100,000 (95% UI: 2498.94–3033.60) to 2499.35 per 100,000 (95% UI: 2236.21–2793.29), with an EAPC of −0.33% (95% CI: −0.37%, −0.29%), showing a similar downward trend in both sexes. The ASMR decreased significantly from 231.78 per 100,000 (95% UI: 198.98–257.42) to 73.23 per 100,000 (95% UI: 59.73–86.85), with an EAPC of −4.25% (95% CI: −4.48%, −4.02%). Women experienced a faster decline (EAPC: −5.00%, 95% CI: −5.28%, −4.72%) compared to men (EAPC: −3.57%, 95% CI: −3.79%, −3.35%). The ASDR dropped from 3852.57 per 100,000 (95% UI: 3349.97–4279.01) to 1227.66 per 100,000 (95% UI: 1048.45–1442.54), with an EAPC of −4.19% (95% CI: −4.38%, −3.99%), and again, the decline in women (EAPC: −4.67%, 95% CI: −4.91%, −4.43%) was faster than in men (EAPC: −3.74%, 95% CI: −3.92%, −3.56%). Despite the continued decline in ASR, the absolute disease burden of COPD has significantly increased: incident cases rose from 2.16 million to 4.43 million, the total number of cases increased from 23.14 million to 50.58 million, and the number of deaths rose from 1.24 million to 1.29 million. Although the total DALYs decreased from 26.09 million to 23.64 million, the overall burden remains high (Figure 1).

**FIGURE 2:**
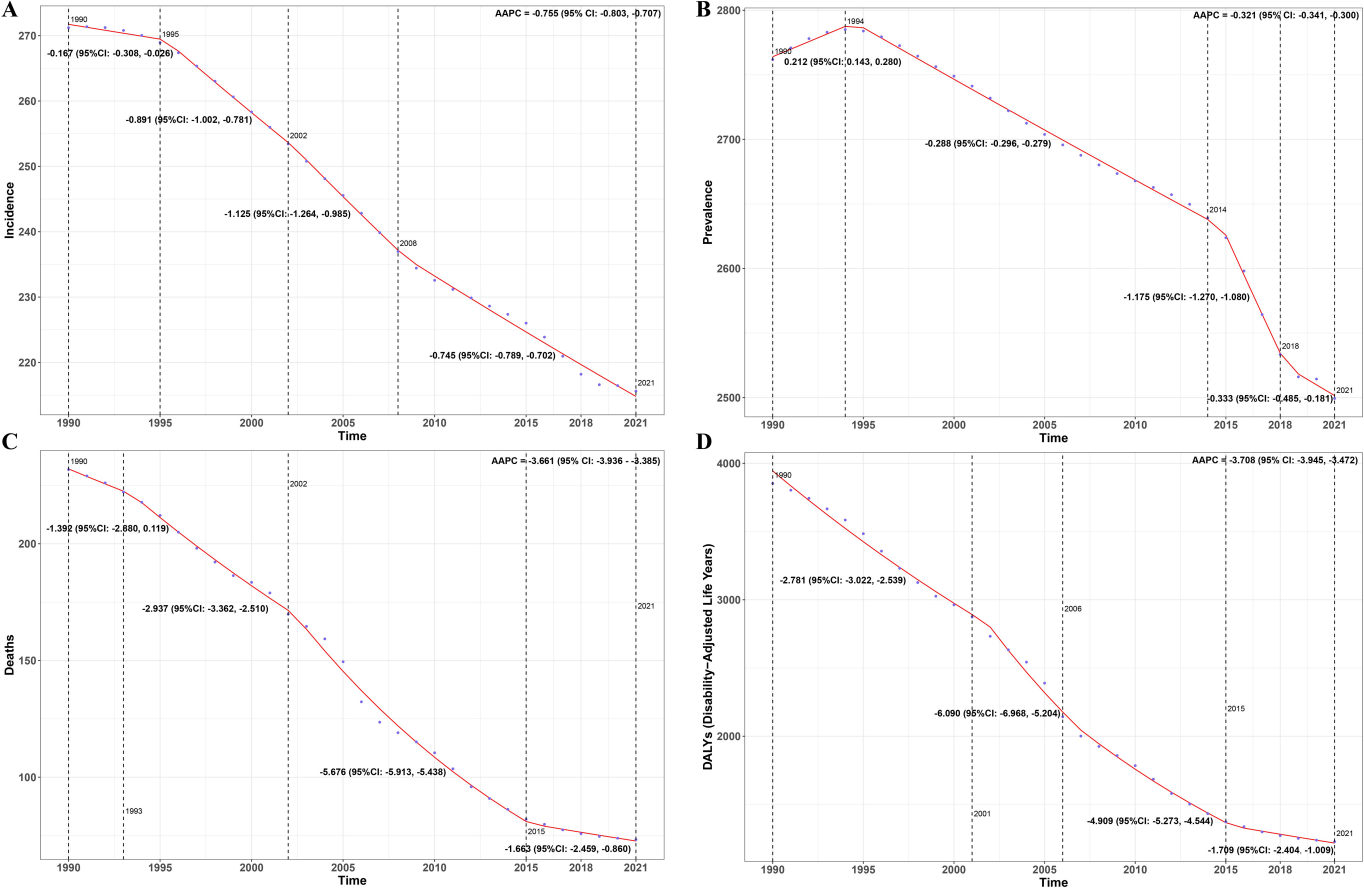
AAPC results [**(A)** ASIR; **(B)** ASPR; **(C)** ASMR; **(D)** ASDR].

Joinpoint regression analysis indicates that between 1990 and 2021, all types of ASR for COPD showed a declining trend. However, the rate of decline in ASIR slowed after 2008, in ASPR after 2018, and in both ASMR and ASDR after 2015 (Figure 2).

### 3.2 Age-sex-temporal trends

**FIGURE 3:**
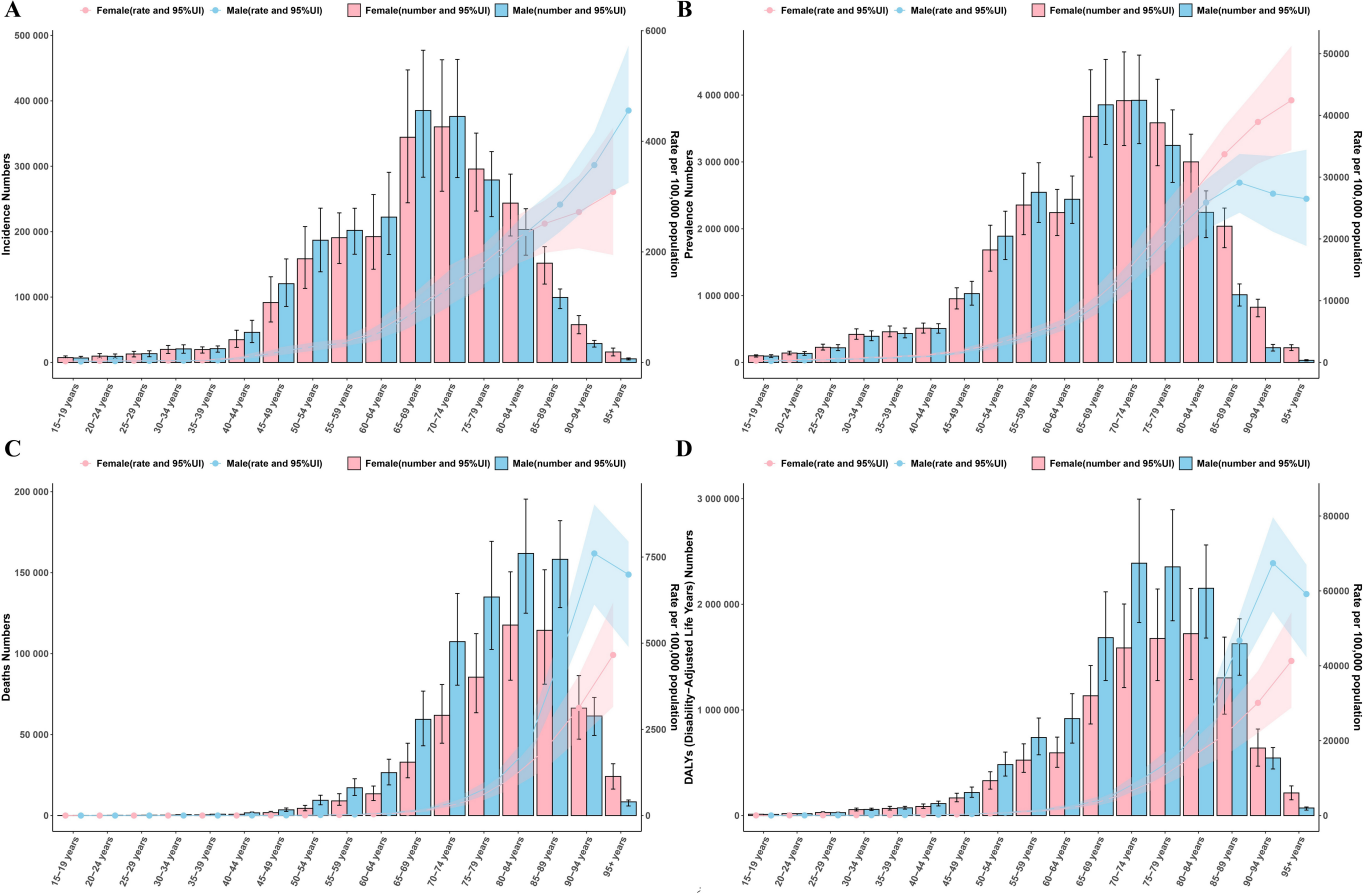
Age-sex analysis results [**(A)** ASIR; **(B)** ASPR; **(C)** ASMR; **(D)** ASDR].

Analysis by age and sex revealed that the ASR for COPD increased with age in both sexes. The male ASPR peaked in the 85–89 age group, while ASMR and ASDR were highest in the 90–94 age group, followed by a slight decline. In women, the ASR continued to increase with age. Specifically, the differences between males and females were minimal before age 60, but after 60, males exhibited higher ASMR and ASDR than females. The absolute number of patients was mainly concentrated in the 60–89 age group (Figure 3). Age and time analysis showed a decline in ASIR, ASMR, and ASDR across all age groups, while ASPR remained relatively stable (Supplementary Figure 1). Time and sex analysis showed a decline in ASR for both sexes, with males consistently showing higher ASIR, ASMR, and ASDR than females. However, since 2015, the gap in ASPR between males and females has gradually narrowed (Supplementary Figure 2).

### 3.3 Decomposition analysis

**FIGURE 4:**
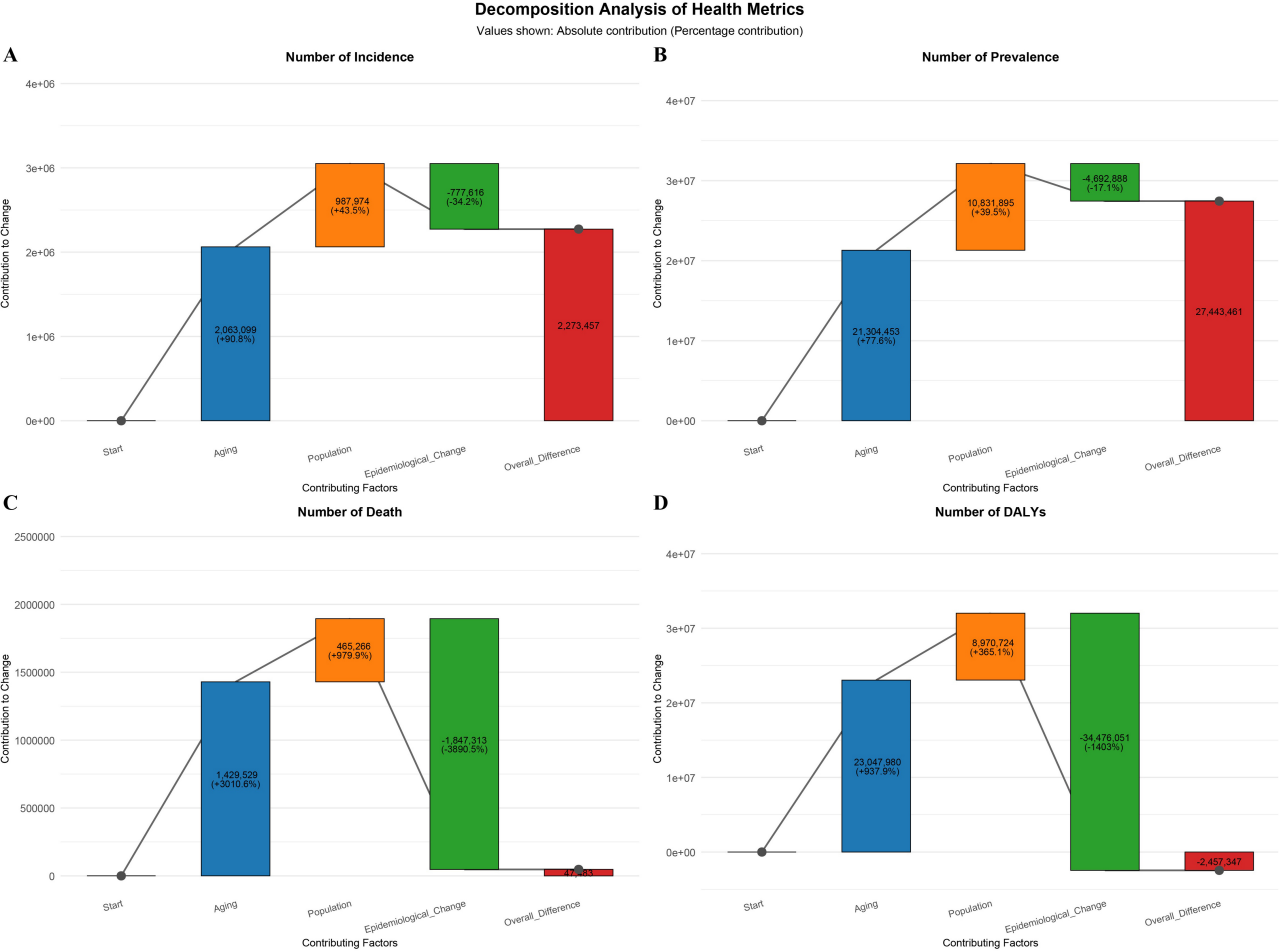
Decomposition analysis results [**(A)** ASIR; **(B)** ASPR; **(C)** ASMR; **(D)** ASDR].

The decomposition analysis showed that population aging was the primary driver of the rising absolute burden of COPD. Its contribution to the increase in incidence was 90.75% (an increase of 2.06 million cases), and to the increase in prevalence, it was 77.63% (an increase of 21.30 million cases). Epidemiological factors offset part of this increase, contributing −34.20% (a reduction of 778,000 cases) to the incidence rate. The net increase in deaths was 47,500, mainly driven by aging (contribution of 3010.63%, an increase of 1.43 million) and population growth (contribution of 979.86%, an increase of 465,000), but this increase was largely offset by improvements in epidemiology (contribution of −3890.49%, a reduction of 1.85 million). In terms of health loss, aging resulted in an increase of 23.05 million DALYs (a + 937.92% increase), while epidemiological improvements led to a reduction of 34.48 million DALYs (a −1402.98% decrease), making it the decisive factor in reducing the health burden (Figure 4).

### 3.4 Predictive analysis

**FIGURE 5:**
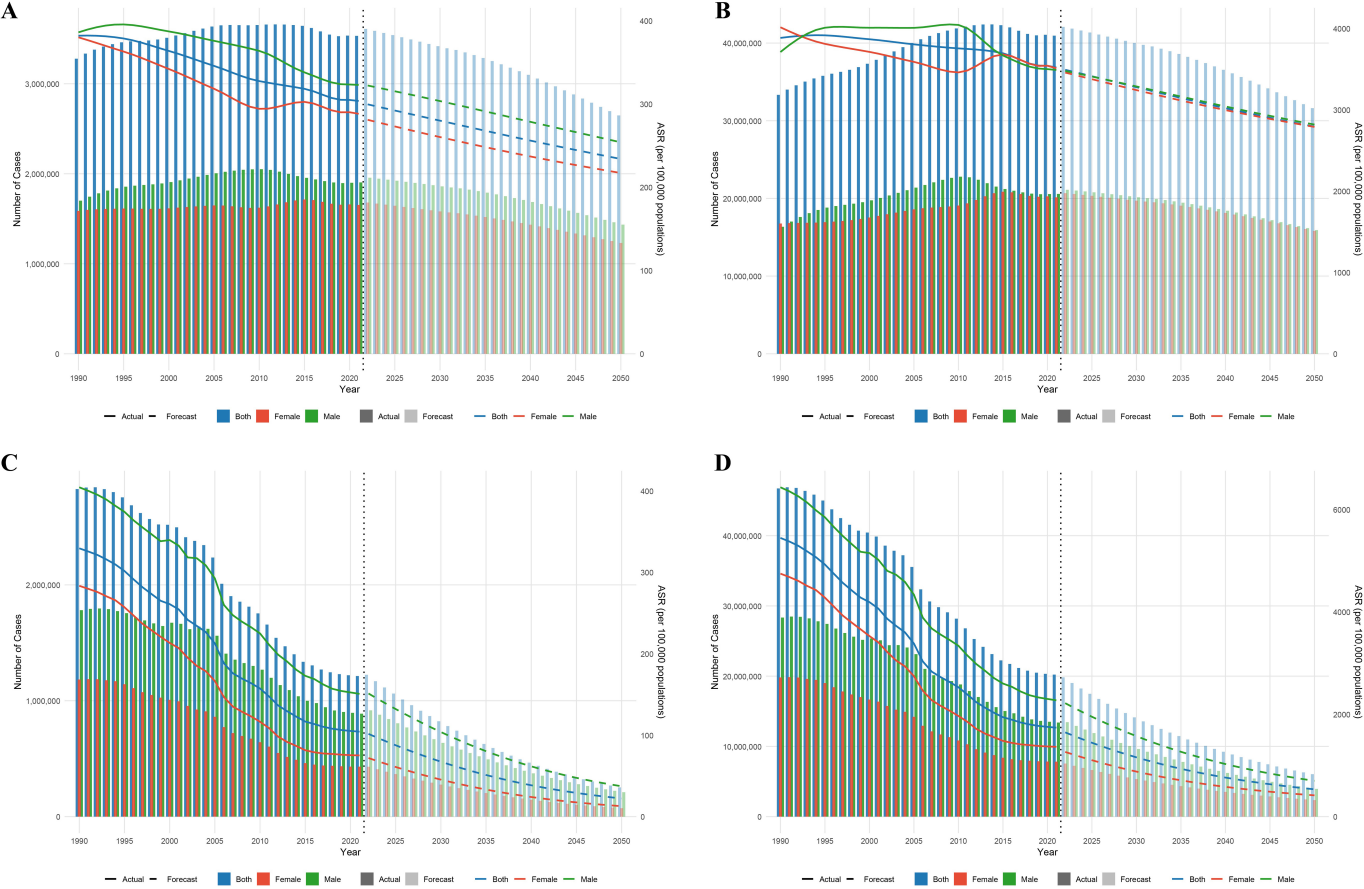
Predictive analysis results [**(A)** Incidence; **(B)** Prevalence; **(C)** Mortality; **(D)** DALYs].

The predictive model suggests that between 2022 and 2050, both the ASR and the absolute number of COPD cases in China will continue to decline, with the disease burden in males remaining higher than in females. By 2050, the expected values are: ASIR: 233.96 (211.21–256.71) per 100,000, ASPR: 2793.42 (2609.80–2977.05) per 100,000, ASMR: 22.30 (14.20–30.40) per 100,000, and ASDR: 533.64 (383.73–683.55) per 100,000 per year (Figure 5 and Supplementary Table 1).

### 3.5 Attribution of risk factors for ASMR

**FIGURE 6:**
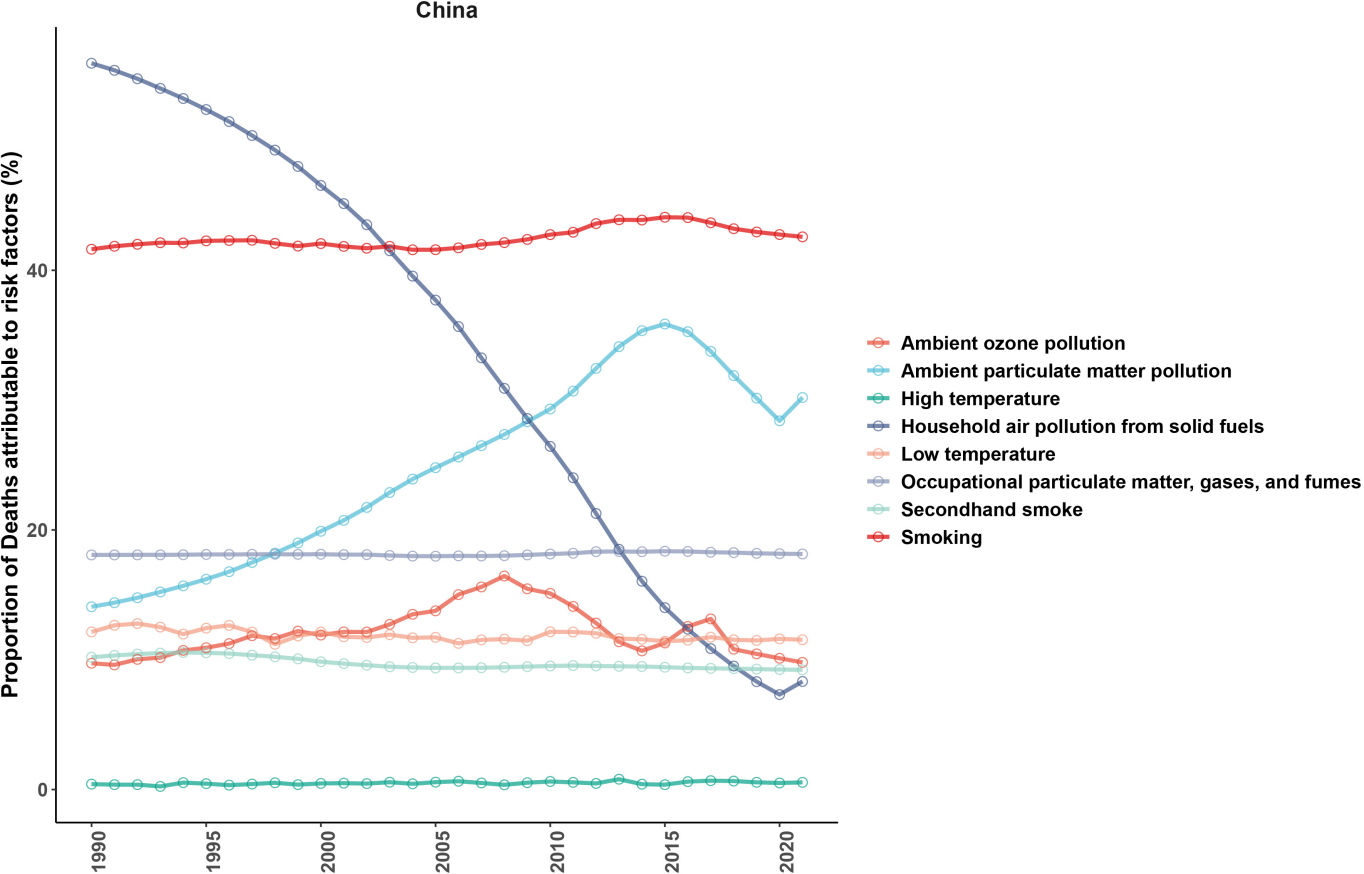
Attributable risk factors for ASMR in COPD.

Attribution analysis revealed that the primary risk factor for COPD is smoking, with the PAF increasing from 41.63% in 1990 to 42.58% in 2021. The PAF for environmental particulate pollution continued to rise, peaking at 35.86% in 2015 (95% CI: 35.27–36.46) before decreasing to 30.20% in 2021. The PAF for indoor solid fuel pollution decreased significantly, from 55.96% in 1990 to 8.32% in 2021, a reduction of 85.10%. The PAF for occupational exposure to particulate matter/gases/smoke remained stable, ranging from 18.07 to 18.35%. The PAF for cold exposure ranged from 11.19 to 12.79%, with 2021 at 11.54%, significantly higher than that for heat exposure (0.24%–0.79%, 2021: 0.55%). The PAF for environmental ozone pollution showed a U-shaped trend, peaking at 16.45% in 2008 and gradually decreasing to 9.80% in 2021. Additionally, the PAF for second-hand smoke steadily decreased from 10.19% to 9.22% (Figure 6).

## 4 Discussion

This study systematically assessed the trends in the burden of COPD in China from 1990 to 2021. The results show that although the ASR have significantly decreased, the absolute burden continues to increase, indicating that China has made progress in addressing COPD, but still faces significant challenges. Predictive analyses further suggest that the ASR will continue to decline in the future, reflecting the positive impact of China’s rapid socioeconomic development on reducing chronic disease risks, including improvements in nutrition, living conditions, educational levels, and healthcare systems (19, 20). In recent years, the government’s increasing attention to COPD and the strengthening of policy support have contributed to this trend. For example, in 2014, COPD monitoring was incorporated into the national chronic disease and nutrition monitoring system, and the “China National Plan for the Prevention and Treatment of Chronic Diseases (2017–2025)” released in 2017 proposed achieving a 25% lung function testing rate by 2025 and controlling the mortality of chronic respiratory diseases (21). The expansion of insurance coverage, advances in diagnostic and treatment technologies, and improvements in basic public health services have also provided a safeguard for reducing mortality (22, 23). Despite the significant decline in COPD ASR, the actual number of patients continues to increase, and APC analysis shows that the rate of decline in ASR has slowed in recent years. This further underscores the significant ongoing challenge that COPD poses to China. Among people aged 40 and above, the awareness rate of COPD is only 0.9%, with awareness of related knowledge and lung function testing at just 5.7 and 3.4%, respectively (24). Therefore, enhancing public knowledge on the prevention and treatment of COPD, along with the widespread implementation of a three-tier prevention strategy, remains a key measure to reduce the COPD burden.

A core finding of this study is the divergence between the decline in China’s COPD ASIR and ASPR from 1990 to 2021, alongside the increasing trend in absolute case numbers. This seemingly contradictory result is actually a classic epidemiological characteristic, primarily driven by significant changes in China’s demographic structure, particularly the rapid aging of its population (25). Age-standardized rates, calculated using a standard population structure, aim to eliminate the confounding effects of differences in the age composition of the population across time periods. Therefore, the decline in both ASIR and ASPR indicates that, after adjusting for the effects of population aging, the true risk of COPD across all age groups in China has decreased, corroborating the positive impact of public health interventions, improved healthcare access, and environmental policies over the past three decades. In contrast, the increase in absolute case numbers is mainly attributed to demographic factors. The risk of developing COPD significantly increases with age (26). Consequently, despite the decline in risk rates across age groups, the sharp expansion of the elderly population (which is already the highest-risk group) has ultimately led to a net increase in the total number of cases. Our decomposition analysis provides precise quantitative evidence for this mechanism: the contribution of population aging to the growth of both incidence and prevalence of COPD is 90.75 and 77.63%, respectively, far exceeding the impacts of population growth and epidemiological changes. This finding underscores the dual challenge faced by China’s healthcare system: while continuing to implement effective risk-reduction strategies, it must also actively address the long-term trend of rising COPD burden driven by population aging.

Further gender-based analysis shows that both male and female ASR values decrease year by year, with the rate of decline faster in women, and the ASR lower in women compared to men. This may be related to the significantly higher smoking rate in men in China (27, 28), as well as their increased exposure to occupational dust (e.g., mineral dust, inorganic dust, metal dust, and grain dust) and chemical irritants (29, 30). Additionally, estrogen may reduce women’s susceptibility through anti-inflammatory and alveolar protective effects, whereas men are more likely to experience environmental damage during critical periods of lung development (31, 32). Compared to men, women generally have better health awareness and healthier lifestyle habits (e.g., appropriate intake of fruits and vegetables, low-salt diet), and exhibit higher treatment compliance, which may contribute to the observed gender differences in COPD (33). Therefore, measures such as smoking cessation, occupational protection, and enhancing health awareness should be adopted to improve men’s health behaviors.

Age-related analysis revealed a significant increase in COPD ASR with age, reflecting the central role of aging in disease progression. Aging, in essence, refers to the decline in the body’s ability to respond to environmental stress, leading to immune aging, reduced airway defense, and loss of lung parenchymal elasticity, all of which increase susceptibility to disease (34, 35). Furthermore, aging interacts with risk factors such as smoking and pollution, accelerating disease progression. This study found that the burden of COPD peaks among the elderly, attributable both to the extension of the exposure window as life expectancy increases in China and the cumulative effect of long-term exposure to tobacco smoke, indoor fuel pollution, and environmental particulate matter (36). Notably, after age 60, male ASMR and ASDR were higher than in females. This primarily reflects the higher prevalence of lung parenchymal damage in men, resulting from lifelong heavy smoking, which accelerates mortality risk, while women tend to have an airway inflammation type of COPD with relatively slower disease progression (37). The absolute number of patients is concentrated in the 60–89 age group, which not only reflects the aging population but also suggests that it typically takes a long latency period for exposure to risk factors to manifest as significant airflow limitation, highlighting the cumulative effect of COPD. It is noteworthy that this study found a significant number of COPD patients in the younger age group of <40 years. This phenomenon may be explained by multiple factors. First, early life is a critical window for lung development, during which exposure to adverse factors (such as maternal smoking, severe lower respiratory tract infections in childhood, malnutrition, and indoor/outdoor air pollution) may hinder the lungs from reaching their full potential. This could lead to individuals exhibiting lower baseline lung function in early adulthood, thus developing airflow limitation earlier in life (38). Second, genetic susceptibility (such as α-1 antitrypsin deficiency or other common genetic variations) may make some individuals more sensitive to environmental exposures, such as tobacco smoke, accelerating the decline in lung function (39). Furthermore, as clinical awareness of COPD increases and diagnostic capabilities improve, more mild or early-stage patients are being identified and diagnosed at younger ages. Although the age-specific prevalence in this group is much lower than in the elderly population, the absolute number of patients remains a significant disease burden, given China’s large population. This finding underscores the critical importance of advancing the prevention window for COPD, suggesting that public health interventions should target childhood and adolescence, aiming to optimize lung development and reduce early adverse exposures, thus delaying or preventing the onset of COPD from the early stages of the life course. Decomposition analysis further quantified the role of aging in COPD, showing that population aging accounted for 90.75% of the increase in incidence and 77.63% of the increase in prevalence, becoming the primary driver of the rising absolute burden. This phenomenon aligns with China’s accelerating aging process. It is worth noting that the net increase in deaths is largely driven by aging (contributing 3010.60%, an increase of 1.43 million) and population growth (contributing 979.90%, an increase of 465,000), far outpacing the expansion of population size, highlighting the impact of cumulative exposure damage, multimorbidity, and immune aging on elderly COPD patients (35). However, significant improvements in epidemiological factors (with contributions ranging from −34.20 to −3890.50%) have been the main factors mitigating the growth of the COPD burden, directly reflecting the effectiveness of China’s anti-smoking campaigns, clean energy policies, and chronic respiratory disease management. Nonetheless, APC analysis indicates that the rate of decline in ASR has slowed in recent years, particularly with ASPR remaining relatively stable, indicating that existing measures have not yet been able to reverse the trend of case accumulation driven by population aging.

Regarding attributable risk factors, smoking remains the primary modifiable risk factor for COPD-related mortality, with its PAF rising from 41.63% in 1990 to 42.58% in 2021. This indicates that despite ongoing tobacco control policies, the large smoking base and cumulative exposure effects continue to cause significant health losses. Tobacco smoke promotes lung tissue damage through mechanisms involving airway inflammation, oxidative stress, and protease–antiprotease imbalance, with men being more prone to the emphysema type of COPD, which carries a higher risk of mortality (37, 40). The PAF for environmental particulate exposure follows an inverse U-shape, peaking in 2008 at 35.86% (95% CI: 35.27–36.46), before decreasing to 30.20% in 2021. Although the concentrations of pollutants like PM2.5 have significantly decreased, urban heat island effects and extreme temperature variations still pose a significant threat to elderly populations (41, 42). The PAF for cold exposure has remained at around 11.50%, significantly higher than that for heat exposure (0.55% in 2021). This difference is attributed to mechanisms such as bronchospasm, impaired mucociliary function, and increased infection risk, particularly in the elderly (43, 44). The PAF for solid fuel use decreased by more than 85%, highlighting the effectiveness of China’s clean energy policies, though rural areas in the western regions remain concentrated sites of residual risk, requiring solutions such as increased clean energy adoption and improved indoor ventilation (45, 46). Occupational exposure has remained stable at around 18%, suggesting that occupational health management still needs improvement, with continued exposure in high-risk industries like mining and construction maintaining high COPD burdens in men (47).

Although this study provides a comprehensive analysis of the COPD disease burden, it has limitations. Firstly, the research is primarily based on national-level data and does not address provincial heterogeneity, which may obscure regional differences. Secondly, the GBD model does not include some potential risk factors (e.g., e-cigarettes, genetic factors) and their interactions, and there is an issue of multiexposure collinearity that may affect the accuracy of the attribution results.

## 5 Conclusion

In conclusion, the ASR for COPD in China significantly declined from 1990 to 2021, with expectations that this trend will continue. However, the rate of decline has slowed in recent years, and the absolute number of patients continues to rise, indicating that COPD remains a significant public health issue. The ASR for both males and females increases with age, with similar rates prior to age 60. After 60, the male ASMR and ASDR are higher than those in females. Population aging is the primary driver of the increased disease burden, while epidemiological changes have reduced the burden. Smoking remains the leading risk factor for COPD-related mortality, and eight other related risk factors have been identified. Future efforts should focus on strengthening smoking cessation, environmental governance, and lung function screening for high-risk populations to enable early diagnosis and treatment, along with the development of targeted health management and medical strategies to reduce the COPD burden. Additionally, it should be considered that GBD estimates may be affected by insufficient reporting of underlying data, misclassification of diagnoses, or inconsistencies in coding, which could reduce the reliability of the results.

## Data Availability

The original contributions presented in this study are included in this article/Supplementary material, further inquiries can be directed to the corresponding author.
