# Supplementary material for: Burden of chronic obstructive pulmonary disease in China from 1990 to 2021: a population-based study
Source: Front Med (Lausanne). 2025 Oct 15;12:1674952. doi: 10.3389/fmed.2025.1674952 (PMC12568032; doi:10.3389/fmed.2025.1674952)
Supplement: Supplementary file 1 [file Table_1.docx]

**Analysis of the Burden of COPD in China from 1990 to 2021: A Population-Based Study**


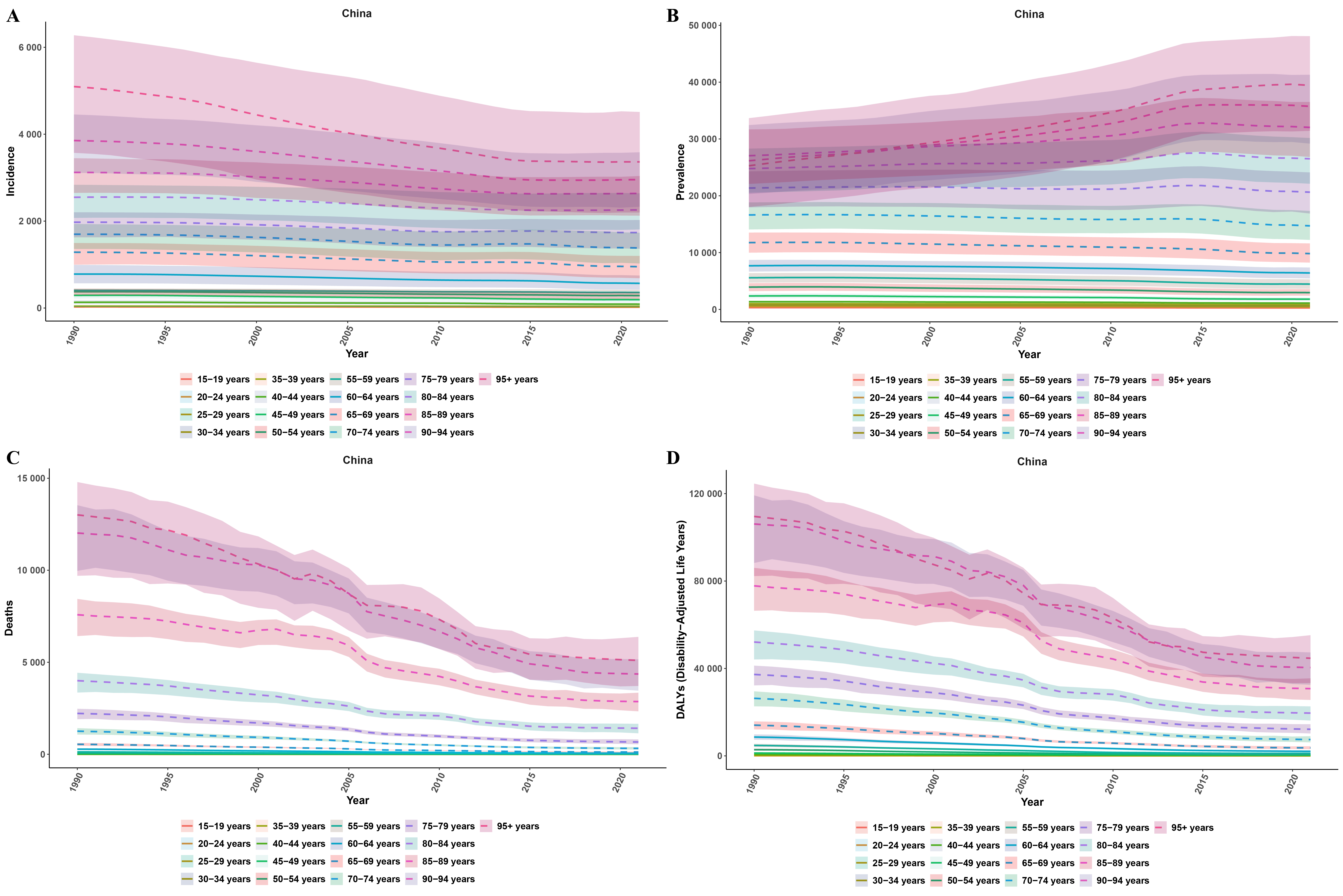


**Supplementary Figure 1: Age-related Temporal Trend**


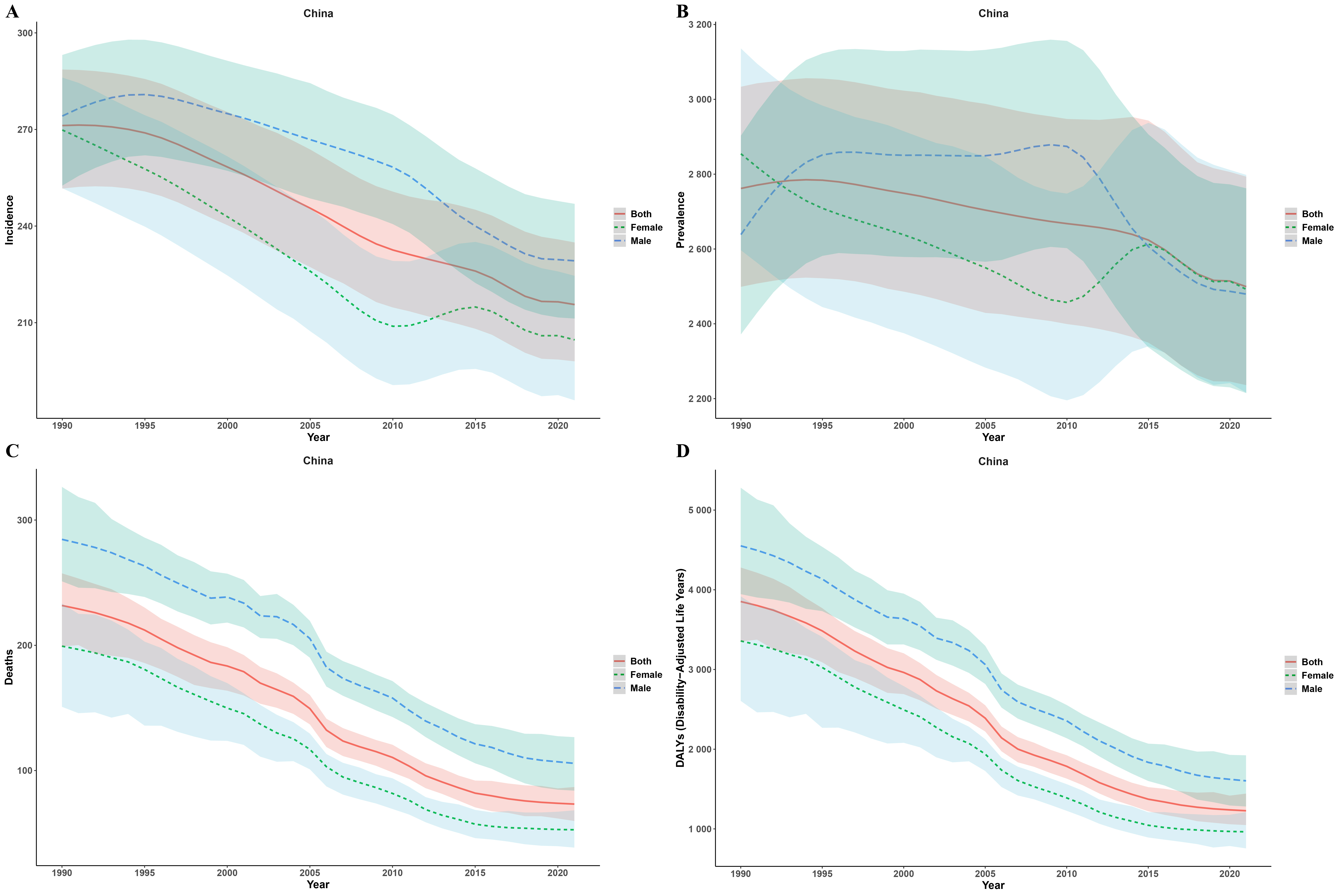


**Supplementary Figure 2: Gender-related Temporal Trend**

**Supplementary Table 1: Predictive Analysis Results**

|  | ASIR | | | ASPR | | | ASMR | | | ASDR | | |
| --- | --- | --- | --- | --- | --- | --- | --- | --- | --- | --- | --- | --- |
| Time | val | low_95 | up_95 | val | low_95 | up_95 | val | low_95 | up_95 | val | low_95 | up_95 |
| 2022 | 299.571 | 291.6537 | 307.4883 | 3492.314 | 3426.148 | 3558.479 | 101.5533 | 95.38634 | 107.7204 | 1645.559 | 1568.015 | 1723.104 |
| 2023 | 297.0919 | 288.3448 | 305.8389 | 3466.151 | 3394.624 | 3537.679 | 96.74813 | 89.0977 | 104.3986 | 1575.023 | 1478.613 | 1671.433 |
| 2024 | 294.6216 | 285.0996 | 304.1437 | 3439.604 | 3362.979 | 3516.228 | 92.1045 | 83.3842 | 100.8248 | 1507.477 | 1397.102 | 1617.851 |
| 2025 | 292.1643 | 281.9095 | 302.4191 | 3412.777 | 3331.262 | 3494.292 | 87.62544 | 78.09784 | 97.15304 | 1442.801 | 1321.54 | 1564.062 |
| 2026 | 289.7187 | 278.7643 | 300.6732 | 3386.054 | 3299.795 | 3472.312 | 83.29094 | 73.14713 | 93.43475 | 1380.755 | 1250.812 | 1510.699 |
| 2027 | 287.278 | 275.6507 | 298.9054 | 3359.312 | 3268.426 | 3450.198 | 79.10005 | 68.48831 | 89.71178 | 1321.142 | 1184.217 | 1458.068 |
| 2028 | 284.8389 | 272.5662 | 297.1117 | 3332.407 | 3237.051 | 3427.763 | 75.0528 | 64.09653 | 86.00906 | 1264.07 | 1121.535 | 1406.604 |
| 2029 | 282.4063 | 269.5133 | 295.2993 | 3305.352 | 3205.675 | 3405.029 | 71.1675 | 59.96729 | 82.36771 | 1209.647 | 1062.612 | 1356.683 |
| 2030 | 279.9778 | 266.4864 | 293.4692 | 3278.272 | 3174.39 | 3382.154 | 67.44301 | 56.08265 | 78.80337 | 1157.866 | 1007.232 | 1308.5 |
| 2031 | 277.5548 | 263.4834 | 291.6261 | 3251.539 | 3143.527 | 3359.551 | 63.87705 | 52.42699 | 75.3271 | 1108.576 | 955.1035 | 1262.049 |
| 2032 | 275.1278 | 260.4927 | 289.763 | 3225.013 | 3112.927 | 3337.099 | 60.45301 | 48.97568 | 71.93034 | 1061.562 | 905.9096 | 1217.214 |
| 2033 | 272.7021 | 257.5208 | 287.8834 | 3198.532 | 3082.464 | 3314.6 | 57.17994 | 45.72905 | 68.63084 | 1016.838 | 859.5729 | 1174.103 |
| 2034 | 270.2834 | 254.5731 | 285.9937 | 3172.116 | 3052.152 | 3292.079 | 54.06448 | 42.68427 | 65.4447 | 974.4523 | 816.0362 | 1132.868 |
| 2035 | 267.8765 | 251.6522 | 284.1007 | 3145.893 | 3022.092 | 3269.694 | 51.1166 | 39.84077 | 62.39243 | 934.3845 | 775.1877 | 1093.581 |
| 2036 | 265.4794 | 248.7546 | 282.2043 | 3120.17 | 2992.555 | 3247.786 | 48.32048 | 37.17872 | 59.46224 | 896.4468 | 736.7915 | 1056.102 |
| 2037 | 263.089 | 245.875 | 280.3031 | 3094.797 | 2963.372 | 3226.221 | 45.65844 | 34.67838 | 56.63851 | 860.3715 | 700.559 | 1020.184 |
| 2038 | 260.7099 | 243.0196 | 278.4002 | 3069.595 | 2934.397 | 3204.793 | 43.12379 | 32.33083 | 53.91676 | 826.0827 | 666.3826 | 985.7828 |
| 2039 | 258.3568 | 240.2021 | 276.5115 | 3044.593 | 2905.649 | 3183.536 | 40.72949 | 30.14064 | 51.31833 | 793.6149 | 634.2338 | 952.9961 |
| 2040 | 256.0351 | 237.4263 | 274.6439 | 3019.907 | 2877.218 | 3162.595 | 38.4826 | 28.10717 | 48.85803 | 762.9956 | 604.0753 | 921.9159 |
| 2041 | 253.7446 | 234.6903 | 272.7989 | 2995.779 | 2849.311 | 3142.246 | 36.37748 | 26.22096 | 46.53401 | 734.1022 | 575.7589 | 892.4455 |
| 2042 | 251.4745 | 231.9821 | 270.9669 | 2972.045 | 2821.747 | 3122.343 | 34.38938 | 24.45982 | 44.31895 | 706.721 | 549.0713 | 864.3707 |
| 2043 | 249.222 | 229.3006 | 269.1435 | 2948.538 | 2794.382 | 3102.693 | 32.51218 | 22.81672 | 42.20765 | 680.7501 | 523.9036 | 837.5966 |
| 2044 | 246.993 | 226.6509 | 267.335 | 2925.282 | 2767.23 | 3083.334 | 30.74614 | 21.28806 | 40.20422 | 656.1715 | 500.2042 | 812.1388 |
| 2045 | 244.7925 | 224.0364 | 265.5485 | 2902.382 | 2740.364 | 3064.4 | 29.09902 | 19.87494 | 38.32311 | 632.9753 | 477.9256 | 788.0251 |
| 2046 | 242.6121 | 221.4476 | 263.7766 | 2880.001 | 2713.913 | 3046.088 | 27.56249 | 18.56778 | 36.5572 | 611.0463 | 456.9461 | 765.1466 |
| 2047 | 240.4423 | 218.8734 | 262.0113 | 2857.985 | 2687.704 | 3028.266 | 26.12249 | 17.35403 | 34.89096 | 590.2304 | 437.1189 | 743.3418 |
| 2048 | 238.2744 | 216.3067 | 260.242 | 2836.184 | 2661.597 | 3010.771 | 24.76739 | 16.22398 | 33.3108 | 570.4246 | 418.3487 | 722.5005 |
| 2049 | 236.1143 | 213.7526 | 258.476 | 2814.635 | 2635.612 | 2993.658 | 23.49453 | 15.17326 | 31.81581 | 551.5756 | 400.5718 | 702.5794 |
| 2050 | 233.9613 | 211.2083 | 256.7142 | 2793.423 | 2609.796 | 2977.05 | 22.29882 | 14.19567 | 30.40197 | 533.6404 | 383.7313 | 683.5495 |
